# Pt-Au Nanoparticles in Combination with Near-Infrared-Based Hyperthermia Increase the Temperature and Impact on the Viability and Immune Phenotype of Human Hepatocellular Carcinoma Cells

**DOI:** 10.3390/ijms26041574

**Published:** 2025-02-13

**Authors:** Marzena Szwed, Tina Jost, Emilia Majka, Nasrin Abbasi Gharibkandi, Agnieszka Majkowska-Pilip, Benjamin Frey, Aleksander Bilewicz, Rainer Fietkau, Udo Gaipl, Agnieszka Marczak, Dorota Lubgan

**Affiliations:** 1 Department of Medical Biophysics, Institute of Biophysics, Faculty of Biology and Environmental Protection, University of Lodz, 90-236 Lodz, Poland; agnieszka.marczak@biol.uni.lodz.pl; 2 Translational Radiobiology, Department of Radiation Oncology, Universitätsklinikum Erlangen, Friedrich-Alexander-Universität Erlangen-Nürnberg, D-91054 Erlangen, Germany; tina.jost@uk-erlangen.de (T.J.); benjamin.frey@uk-erlangen.de (B.F.); udo.gaipl@uk-erlangen.de (U.G.); dorota.lubgan@uk-erlangen.de (D.L.); 3 Comprehensive Cancer Center Erlangen-EMN, D-91054 Erlangen, Germany; rainer.fietkau@uk-erlangen.de; 4 Department of Radiation Oncology, Universitatsklinikum Erlangen, Friedrich-Alexander-Universität Erlangen-Nürnberg, D-91054 Erlangen, Germany; 5 Institute of Nuclear Chemistry and Technology, 03-195 Warsaw, Poland; e.majka@ichtj.waw.pl (E.M.); n.abbasi@ichtj.waw.pl (N.A.G.); a.majkowska@ichtj.waw.pl (A.M.-P.); a.bilewicz@ichtj.waw.pl (A.B.); 6 Deutsches Zentrum Immuntherapie, D-91054 Erlangen, Germany; 7 FAU Profile Center Immunomedicine (FAU I-MED), Friedrich-Alexander-Universität (FAU) Erlangen-Nürnberg, D-91054 Erlangen, Germany

**Keywords:** nanoparticles, hepatocarcinoma, immune checkpoint molecules, NIR, hyperthermia

## Abstract

Near-infrared light (NIR)-responsive metal-based nanoparticles (NPs) could be used for tumour therapy. We examined how platinum (Pt), gold (Au), and core-shell Pt-Au NPs affect the viability of human hepatocellular carcinoma (HCC) cell lines (Hep3B, HepG2, and Huh7D-12) alone and in combination with NIR exposure. In addition, the expression of immune checkpoint molecules (ICMs) on the tumour cells was analysed. We revealed that the cytotoxicity and programmed cell death induction of Au and Pt-Au NPs toward HCC cells could be enhanced by NIR with 960 nm in a different way. Pt-Au NPs were the only particles that resulted in an additional temperature increase of up to 2 °C after NIR. Regarding the tumour cell immune phenotype, not all of the cells experienced changes in immune phenotype. NIR itself was the trigger of the alterations, while the NPs did not significantly affect the expression of most of the examined ICMs, such as PD-L1, PD-L1, HVEM, CD70, ICOS-L, Ox40-L, and TNFRSF9. The combination of Pt-Au NPs with NIR resulted in the most prominent increase of ICMs in HepG2 cells. We conclude that the thermotherapeutic effect of Pt-Au NP application and NIR could be beneficial in multimodal therapy settings in liver cancer for selected patients.

## 1. Introduction

Liver cancer is the fifth most common cancer and the third most common cause of cancer-related death in the world [1]. The diagnosis of hepatocellular carcinoma (HCC) is highly linked with the stage of disease and is often delayed due to a lack of symptoms and patients’ awareness. In parallel, tumour staging plays an essential role in guiding treatment decisions, but prognosis is affected by the severity of underlying liver dysfunction. Patients in the early stages have a much higher chance of curative response with different treatment options [2,3]. The most prominent standard of care that happens during the early or intermediate stages of HCC is liver transplantation. If it is not feasible, other treatment approaches may include surgical tumour resection, transarterial chemoembolization, or radioembolization. Additional therapy commonly used for advanced HCC includes broad-spectrum tyrosine kinase inhibitors, such as Sorafenib and Lenvatinib, as well as a combination of immunotherapy and anti-angiogenesis therapy [4,5]. These strategies offer a nominal extension of the survival curve, but they come with broad-spectrum toxic side effects, ultimately leading to patients developing resistance to therapy. Additionally, the risk of HCC recurrence rates remains as high as 70% within 5 years after curative treatment, and there is an urgent need for new adjuvant treatment options [6,7].

Currently, the nanoparticle (NP)-based liver cancer treatment strategies have significant advantages, such as reducing therapy-related toxicity and increasing the possibility of precise drug delivery [8]. NPs are a unique material that, depending on their properties, such as small size, large surface area to mass ratio, and high reactivity, commonly accumulate in the liver after systemic injection [7]. Additionally, specificity in the liver to the cancer cells can be ensured by the passive targeting utilization of the enhanced permeability and retention effect. Consequently, small-sized NPs can accumulate more effectively in the tumour than in healthy, normal tissues [9].

Another approach in multimodal anticancer therapy, currently also under intensive development, is hyperthermia (HT) generated by an alternating magnetic field, radiofrequency, lasers, or ultrasounds. The basis of this method relies on the fact that elevated temperatures localized within the tumour can damage and/or kill cancer cells, particularly when combined with radio- or chemotherapy [10].

HT is usually considered at two different temperature ranges, in which the increased temperature results in different cellular damage [11]. Moderate temperature (40–44 °C) has a direct influence on secondary and tertiary protein structures, which, following heat exposure, formulate aggregates and denaturize. This results in protein complex deposition, enlarged nuclei formation, and a decrease in the number of mitochondria and lysosomes. In parallel, supraphysiological temperature growth inhibits DNA replication and transcription as well as disrupts mRNA processing [12]. Further, immune activation can result from various HT-mediated mechanisms [10]. Different practices of HT application in multimodal clinical settings exist in Europe [13].

On the other hand, the cells treatment at temperatures above 46 °C, defined as thermal ablation [14], leads to the destruction of cancer tissue from severe and short-term HT. Generally, in thermal ablation therapy, the main cellular death mechanism is coagulative necrosis [15,16] (due to a dramatic temperature increase above 60 °C), which corresponds to serious damage and inflammation [17]. This suggests that HT not only kills cancer cells but again also stimulates the immune system. For example, heat shock proteins impact the innate and adaptive immune system by boosting the production of proinflammatory cytokines, which activate antigen-presenting cells (APCs), such as dendritic cells (DCs) and macrophages [18,19]. In addition, HT impacts the expression of immune-modulating immune checkpoint molecules (ICMs) [20]. Inhibitory and stimulatory immune checkpoint molecules are defined as pairs of ligand receptors that produce either repressing (PD-L1, PD-L2, and HVEM) or stimulating (CD70, ICOS-L, OX40-L, and TNFRSF9) effects on the immune response. Stimulatory checkpoint molecules may boost anti-tumour immunity and thus have begun to be explored in immunotherapy. On the other hand, inhibitory checkpoint molecules are beneficial in preventing autoimmunity but detrimental in the context of anticancer therapy [21]. In this context, a novel application of HT should be enriched by a precise characterization of cancer cells’ immune phenotype and a complete identification of patients who respond well to this type of treatment [22].

Even though magnetic HT is already being used in several clinical trials, there has been an increasing focus on HT induced by photothermal therapy (PT), where heat is generated through near-infrared (NIR) light that has greater tissue transparency in comparison to the laser light in a visible spectrum. Additionally, NPs can preferably absorb energy originating from an NIR external source and focus the heat on the tumour. Thus, it is possible to induce the localized thermal destruction of cancer cells while minimizing the adverse effects on collateral tissue [23].

The literature data describe many other cases that focus on the application of liposomes or dendrimers and gold NPs (Au NPs) on Nano-Photo-Thermal Therapy [24]. Au NPs with unique properties, such as small size, high biocompatibility, low toxicity, and versatility due to the ease of surface functionalization, are highly versatile in a range of medical applications. In drug delivery, Au NPs can be loaded with therapeutic agents and used to target specific cells or tissues in the body. By modifying the surface of gold nanoparticles with targeting molecules (such as antibodies or peptides), drugs can be delivered directly to diseased areas, such as cancerous tumours, minimizing damage to healthy tissues and improving treatment efficacy [25,26,27].

In direct cancer treatment, AuNPs are studied for their potential in photothermal therapy, where they are directed at tumour cells and then heated using near-infrared light. The nanoparticles absorb the light, converting it into heat, which can destroy cancer cells while sparing surrounding healthy tissue [28]. Au NPs are also used in imaging [29]. Due to their ability to scatter light and be easily modified, gold nanoparticles are utilized in imaging techniques like surface-enhanced Raman scattering (SERS) and optical imaging. They can also enhance contrast in imaging modalities such as X-ray or MRI, improving the detection of diseases. Recently, Au NPs have also been explored as vectors for gene delivery, as they can carry DNA or RNA into cells. This could be useful for treating genetic disorders or for cancer immunotherapy, where genetic material is introduced to enhance immune response [30].

In addition, the structure modification of AuNPs, for instance, by using different noble metals may alter NPs sensitivity to NIR light [31]. For instance, platinum ions from the Pt nanoparticles, in combination with Au NPs, could be used as anticancer therapeutics with an effect similar to cisplatin [32]. Moreover, due to high extinction coefficients in the NIR region of Au NPs and Pt NPs, these particles can be used in photothermal therapy [33]. Depciuch et al. suggested that PtAu nanoraspberries (NRs) could be applied as effective light-absorbers in PTT anticancer therapy. High values of these nanoparticles’ photothermal efficiency, 72% for the 650 nm laser and 60% for the laser with 808 nm wavelength, make them good candidates for PTT anticancer therapy [34].

Considering the photothermal conversion effect of nanoparticles after NIR exposure and its potential in anticancer therapy, we asked whether platinum and gold-based NPs could be used as a tool for hyperthermia induction in HCC in vitro. The objective of the present study was to investigate how “nano-hyperthermia” affects the viability and immune phenotype of human HCC cell lines (HepG2, Hep3B, Huh-7D 12). We thereby checked if the NPs 2 nm Pt, 30 nm Au, and core-shell 30 nm Pt-Au after 1 h exposure of NIR with the 960 nm (Hydrosun^®^750) could induce a temperature rise in the tumour cells.

## 2. Results

### 2.1. Physiochemical Characterization of NPs

**Figure 1:**
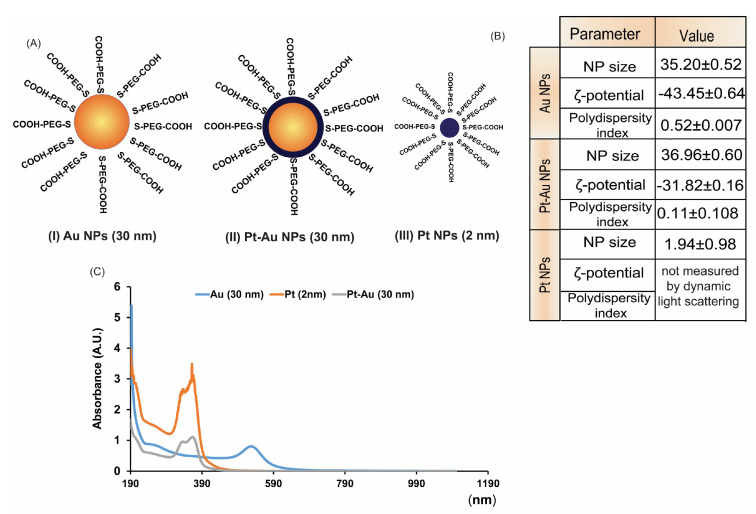
Physico-chemical characterization of noble metal nanoparticles used in this study: (**A**) schematic representation of the NPs: Au NPs (I) Pt-Au NPs (II), and Pt NPs (III); (**B**) particle size, polydispersity index, and zeta potential of the investigated noble metal NPs; (**C**) UV/Vis absorption spectra of Pt, Au, and Pt-Au NPs.

Transmission electron microscopy (TEM) and high-resolution transmission electron microscopy HR-TEM images were present in our previous works [35]. As measured from TEM images, the investigated AuNPs had a mean diameter around 15 nm, Pt-Au 18 nm, and small Pt nanoparticle’s mean diameter was 1.94 ± 0.38 nm (Figure 1A,B).

Spherical shape and high dispersity were also confirmed during imaging. The HR-TEM image presented in our previous work [35] showed a relatively uniform deposition of platinum atoms on the AuNP surface. Measurements of platinum concentration using inductively coupled plasma mass spectrometry (ICP-MS) indicated the presence of two to three layers of Pt on the surface of the gold nanoparticles (Au NPs). The hydrodynamic diameter measured by DLS increased to 35.2 nm for AuNPs and 36.9 for Pt-Au NPs. To ensure stabilization in solution, all synthesized nanoparticles were PEGylated by adding a molar excess of HS-PEG-COOH (5 kDa). The significant change in hydrodynamic diameter and zeta potential after the PEGylation process confirmed the attachment of polyethylene glycol (PEG) molecules to the nanoparticle surface [36]. The stability of the obtained nanoparticle suspension, measured by dynamic light scattering (DLS), showed no change in the hydrodynamic diameter of pegylated nanoparticles during 24 h, indicating that there is no agglomeration. Unfortunately, we could not detect ultrasmall 2 nm platinum (Pt) nanoparticles using DLS. However, the absence of any registered nanostructures larger than 5 nm (which could be detected by DLS) further confirms that there is no agglomeration occurring. All further studies were conducted on pegylated nanoparticles. In parallel, the UV-Vis spectra were drawn, and the maximum absorptions were 362, 370, and 541 nm for Pt-Au, Pt, and Au NPs, respectively.

### 2.2. Following the Exposition to NIR Light Only Pt-Au NPs, Induced a Temperature Increase in Cellular Growth Condition

**Figure 2:**
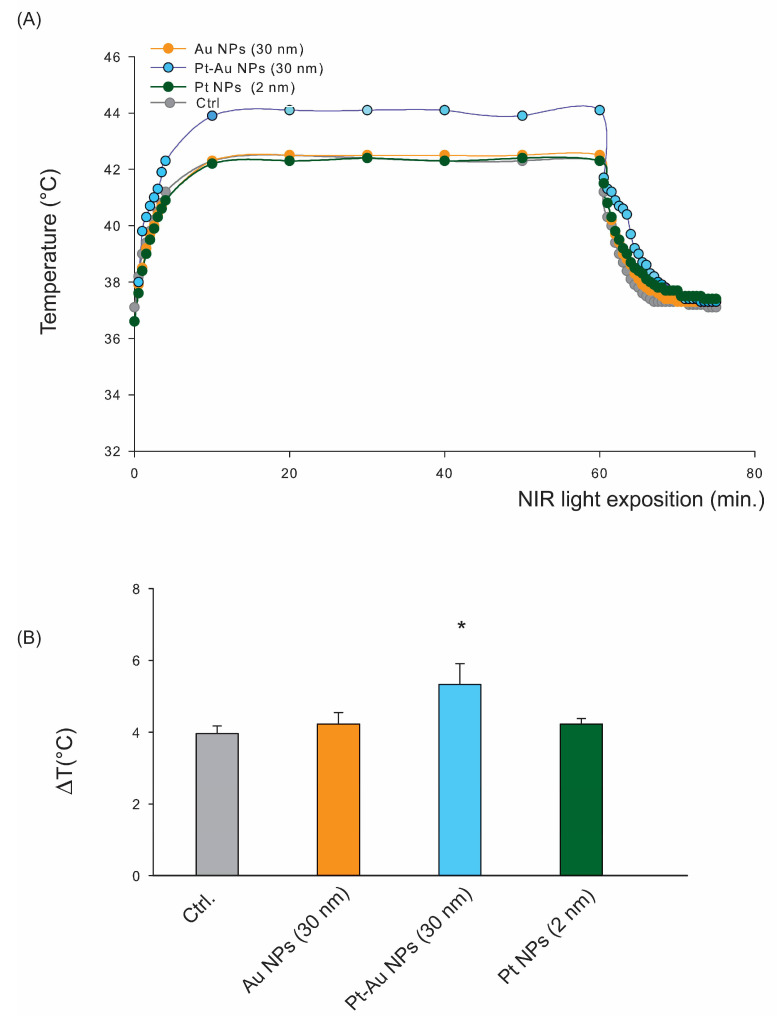
The monitoring of temperature alterations in the experimental setup. (**A**) The example temperature curves of samples containing hepatocellular carcinoma cells alone or with Pt, Au, or Pt-Au NPs during 1 h of NIR exposure. (**B**) Temperature growth in the tested samples (in the presence of Pt, Au, or Pt-Au NPs (55.6 µg/mL) or without NPs) during the first 10 min of NIR exposition, * *p* < 0.05 the statistically significant changes (calculated by using the *t*-test (with Bonferroni correction) between cells with and without NIR exposition.

We focused on NIR radiation-based HT at 225 mW/cm^2^ and 45 cm of distance between the NIR source and the human-derived hepatoma cancer cells in vitro. Au, Pt-Au, and Pt NPs (55.6 µg/mL) in a complete culture medium were simultaneously exposed to NIR (960 nm) at a power density of 38.9 mW/cm^2^. We observed a rapid temperature increase (for up to 44.1 °C) in the cases of Pt-Au NPs, compared to the control conditions (Ctrl), i.e., when cells were cultured without treatment with any particles (Figure 2A). The most significant temperature increase was reached if cells treated with Pt-Au NPs were exposed to NIR for up to 10 min (Figure 2B). However, this phenomenon was observed only for NPs with a gold core and external Pt layer because neither Pt NPs nor Au NPs themselves trigger an increase of temperature in cell culture conditions. Subsequently, after continuous irradiation, the temperature reached a plateau and decreased dramatically when the NIR source was removed. Our data demonstrate that Pt-Au NPs are promising NIR light-to-heat converters with good heat transfer to the culture medium.

### 2.3. A Combination of Pt-Au or Au NPs with NIR-Light Induces Higher Toxicity in Human Hepatoma Cancer Cells Compared to NPs Alone

**Figure 3:**
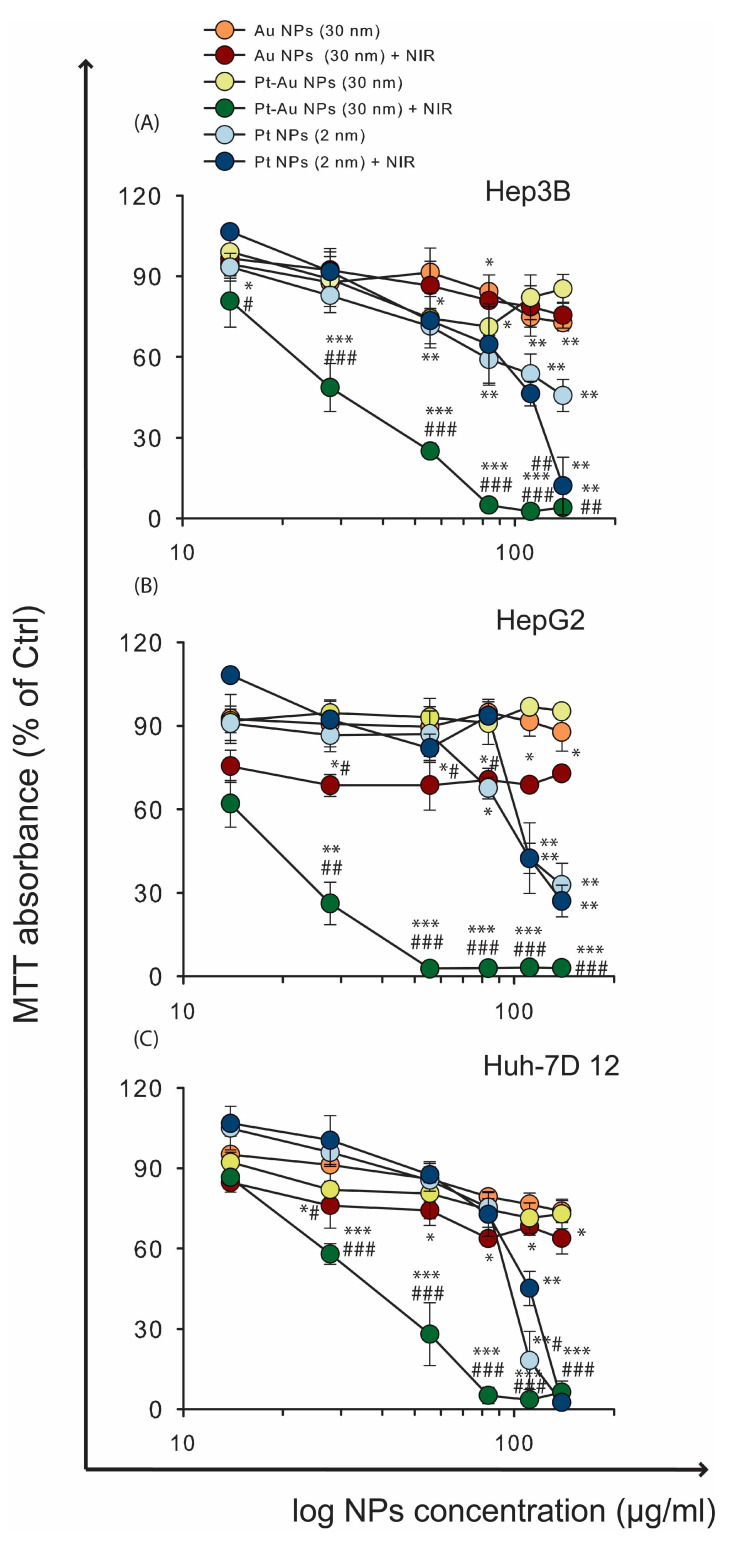
The viability of Hep3B, Huh-7D 12, and HepG2 cells (**A**–**C**) was measured with the MTT assay after 48 h of incubation of cells with increasing concentrations of NPs (0–139 µg/mL) with and without 1 h of NIR exposition. Data are shown as percentage of control cells without or with NIR exposition (cells untreated with NPs and without NIR irradiation or cells untreated with NPs but exposed to NIR light) and represent mean values ± SD of three independent experiments. The statistical significance is calculated using a two-way ANOVA and Tukey’s post hoc analysis. * *p* < 0.05, ** *p* < 0.01, *** *p* < 0.001 statistically significant changes compared to untreated control cells; # *p* < 0.05, ## *p* < 0.01, ### *p* < 0.001 significant differences between probes treated with Au NPs, Pt-Au NPs, and Pt NPs, after exposure to NIR light, and samples incubated only with examined particles.

To assess the cytotoxicity of NPs (in a concentration range of 0–139 µg/mL) and NIR-based hyperthermia in human hepatoma tumour cells, an MTT assay was performed, and cell viability was evaluated 48 h after treatment with NPs alone or in combination with NIR irradiation (Figure 3).

When HCC cells were treated only with Pt NPs (without NIR irradiation), we observed reduced cell viability, and the Huh-7D 12 cell line was the most sensitive to the treatment with these NPs, whereas HepG2 cell cultures displayed comparably lower cytotoxicity. On the contrary, Au NPs or Pt-Au NPs, treated solely, reduced the metabolic activity of Hep3B and Huh-7D 12 cells by up to 80%, while under the same treatment conditions, the changes in HepG2 cell survival rates were barely noticed. When a combination of NP treatment and NIR irradiation was applied, Pt-Au NPs induced increased toxicity in all human hepatoma cancer cells. For instance, when we used 83 μg/mL of NPs and exposed the cells to NIR light, the metabolic activity of the cells decreased drastically to 2%. At lower <83 μg/mL NPs’ concentrations, a striking difference was observed between the examined cell lines. As shown in Figure 3, the HepG2 hepatoma cell line was consistently more sensitive to the application of NPs and NIR exposition compared to incubation with Pt-Au NPs alone. HepG2 cells were 34 times more sensitive to the combination of NIR exposition after 55.6 μg/mL of Pt-Au NPs (*p* = 0.00015). This effect was not so pronounced but still significant either for Hep3B or in Huh-7D 12 cell lines. In addition, HepG2 cells showed increased sensitivity to Au NPs when the incubation with NPs was performed after NIR irradiation. We observed a 20% difference among all tested NP concentrations used together with NIR irradiation. Due to these differences in sensitivity between investigated hepatoma cell lines, we decided to select the dose of 55.6 μg/mL of NPs for our further experiments. Interestingly, NIR applied together with Pt NPs (139 μg/mL) showed the highest toxicity only towards the Hep3B cell line.

When we compared the separate effect of single NIR irradiation without NP treatment, there was a striking difference between the examined cell lines (Figure S1A). The Hep G2 cell line was also the most sensitive here, and the absorbance value was 1.43 times lower in comparison to cells cultured without NIR exposure. However, neither Huh-7D 12 nor Hep 3 B cells displayed decreased mitochondrial activity only after NIR irradiation.

### 2.4. Morphology of Hepatocellular Carcinoma Cells Treated with NPs and Exposed to NIR

**Figure 4:**
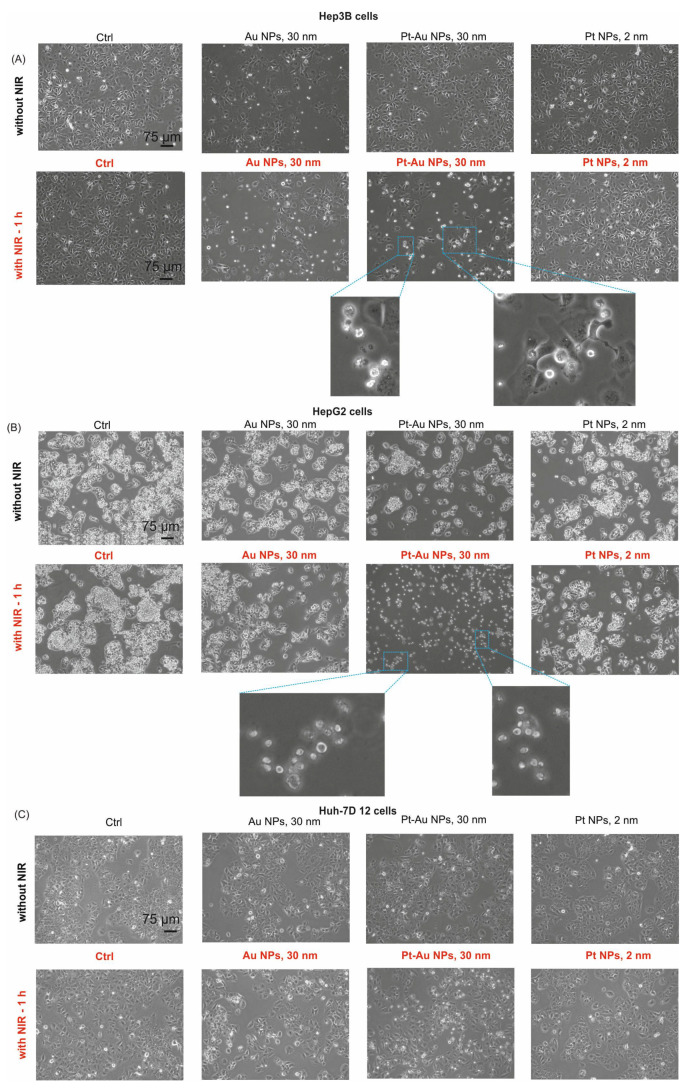
Morphological changes in Hep3B (**A**), HepG2 (**B**), and Huh-7D 12 cells (**C**) visualized by inverted phase contrast microscopy after treatment of cells for 48 h with noble metal NPs in the concentration of 55.6 μg/mL with or without NIR exposition. The bar shown is 75 μm. The zoomed photos show clumped and shrunken cells.

The morphological changes of the tested human hepatocellular carcinoma cell lines, induced by 55.6 µg/mL of Au, Pt-Au, or Pt NPs, with or without the NIR irradiation, were examined after 48 h of incubation under cellular growth conditions. Cultured cells were analysed by inverted light microscopy. As shown in Figure 4, no noticeable changes were observed when the examined NPs were used alone. However, when the additional exposition to the NIR light was carried out, the cells showed the characteristic hallmarks of cellular homeostasis disorders (perinuclear clumping of the cytosol as well as protrusions and extensions from the plasma membrane). These morphological alterations were much more noticeable in the case of HepG2 cell cultures. Furthermore, under the same experimental conditions, a reduction in the number of cells per well was observed, accompanied by an increase in the fraction of cells rounding up. Additionally, to assess the direct effect of NIR light exposure, we took photos of cells immediately after 1 h of NIR irradiation (without NP treatment). Interestingly, no NIR effect was observed at this time (Figure S1B).

### 2.5. Effect of NPs Combined with NIR Light Irradiation on Cell Death Forms

**Figure 5:**
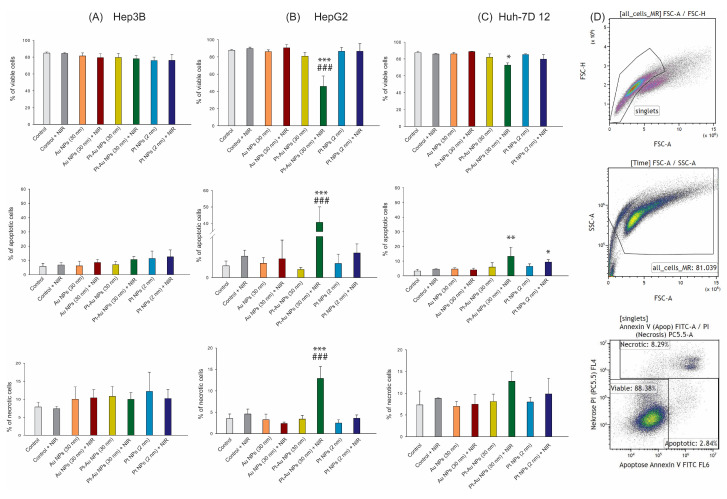
The changes in cell death rates of hepatocellular carcinoma cells treated with noble metal NPs (55.6 µg/mL) with or without NIR exposition. (**A**–**C**): Cell death analyses of the HCC cell lines; Hep3B (**A**), Huh-7D 12 (**B**), and HepG2 (**C**) are displayed as stacked bars that show the mean ± SD, n ≥ 3. (**D**) Gating strategy for the detection of cell death forms by Annexin V/PI staining. Exemplarily shown are data of Hep3B untreated cancer cells. After pre-gating on singlets and then excluding the debris, the remaining cells were identified as viable (Annexin V-, PI-), apoptotic (Annexin V+, PI-), or necrotic (Annexin V+, PI+). The statistical significance is calculated using a two-way ANOVA and Tukey’s post hoc analysis. * *p* < 0.05, ** *p* < 0.01, *** *p* < 0.001 statistically significant changes compared to the untreated control cells; ### *p* < 0.01 significant differences between probes treated with Au NPs, Pt-Au NPs, and Pt NPs, after the exposure to NIR light, and samples incubated only with examined particles.

Annexin-V staining was used to identify phosphatidylserine in the outer leaflet of the cell membrane, an indicator for apoptotic cell death. To analyse cell membrane integrity, cells were additionally labelled with PI, and examples of dot-blots for cell death from analyses as well as the gating strategy are presented in Figure 5D. Tested hepatoma cancer cells were irradiated to NIR light following the treatment with Au, Pt-Au, or Pt NPs (56 μg/mL) and incubated for up to 48 hr. In two out of the three examined tumour cell lines (HepG2 and Huh-7D 12), the combination of Pt-Au NPs with NIR resulted in significant increased apoptosis and, in the case of HepG2 cells, also necrosis. For instance, the fraction of the viable cells decreased significantly to 45% in HepG2, which corresponded to a 1.8-fold drop in reference to the treatment with Pt-Au NPs alone (Figure 5B). In Hep2G cells, the percentage of apoptotic cells reached 40.6, whereas the number of necrotic cells increased 6 times for up to 13% compared to the cell cultures incubated only with Pt-Au NPs and irradiated with NIR light. As shown in Figure 5A, Hep3B cells were the most resistant to the investigated experimental conditions, and NIR exposure did not cause any additional changes in the apoptosis or necrosis induction.

### 2.6. Analyses of Immune Checkpoint Molecules on Hepatoma Cancer Cells Following NIR-Based Hyperthermia in Combination with NP Treatment

**Figure 6:**
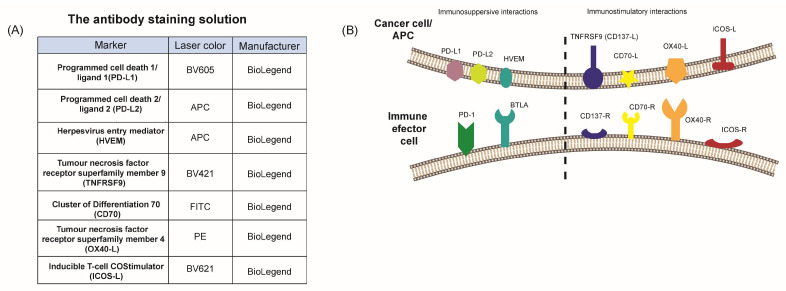
Antibodies and rationale for analyses of expression of immune checkpoint molecules. (**A**) Antibodies used for analyses of the surface expression of immune checkpoint molecules by multicolor flow cytometry. (**B**) Representative profile of ICMs analysed in this study, with relevant specific ligands and receptors expressed on antigen-presenting cells or tumour cells and T cells, respectively. Abb. APC, antigen presenting cell; PD/PD-L1, -L2, programmed cell death 1/ligand 1, 2; HVEM, Herpesvirus entry mediator; BTLA, B- and T-lymphocyte attenuator; TNFRSF9 (CD137), tumour necrosis factor receptor superfamily member 9; CD70-L/R, cluster of differentiation 70; OX40-L/R, tumour necrosis factor receptor superfamily, member 4; ICOS-L/R, Inducible T-cell COStimulator.

Based on cell death induction by NPs and NIR, we additionally examined how the treatment impacted the expression of immune regulatory immune checkpoint molecules on the hepatocellular carcinoma cells (Figure 6B).

**Figure 7:**
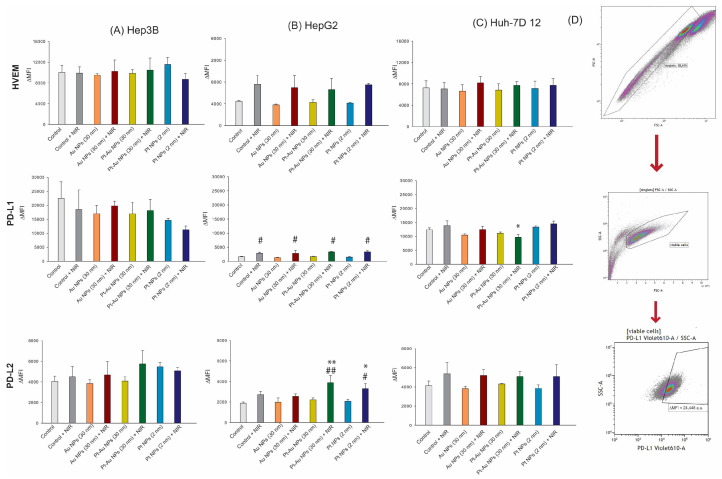
The expression of inhibitory immune checkpoint molecules of HCC cells treated with noble metal NPs (55.6 µg/mL) independently or in combination with NIR-based HT. (**A**–**C**). The expression of inhibitory ICMs (PD-L1, PD-L2 and HVEM) was analysed by multicolor flow cytometry. The mean fluorescence intensity (ΔMFI ± SD, n ≥ 3) is presented from at least five independent experiments. (**D**) Viable cell gating strategy (based on their size and granularity) for the consecutive analysis of inhibitory immune checkpoint molecules on the cell surface of hepatoma tumour cells. The statistical significance is calculated using a two-way ANOVA and Tukey’s post hoc analysis. * *p* < 0.05, ** *p* < 0.01, statistically significant changes compared to untreated control cells, # *p* < 0.05, ## *p* < 0.01 significant differences between probes treated with Au NPs, Pt-Au NPs, and Pt NPs, after exposure to NIR light and samples incubated only with examined particles.

**Figure 8:**
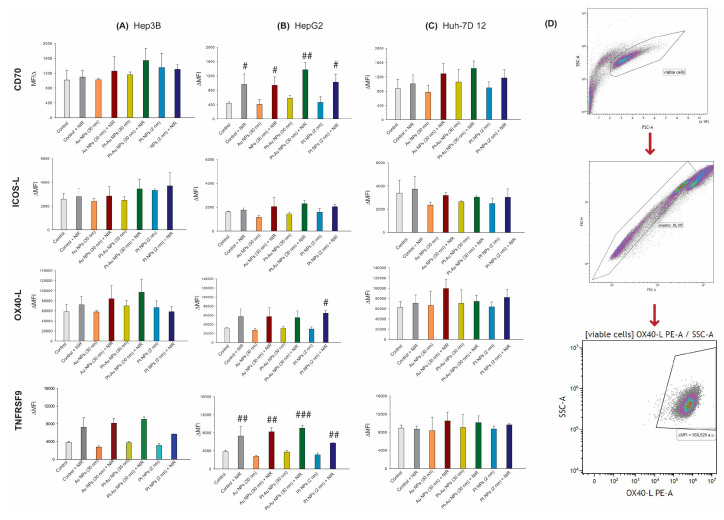
The expression of stimulatory immune checkpoint molecules of HCC cells treated with noble metal NPs independently or in combination with NIR-based HT. (**A**–**C**) For the HCC cells treated with noble metal NPs with or without NIR exposition, ΔMFI of the stimulatory immune checkpoint molecules CD70, ICOS-L, OX40-L, and TNFRSF9 are shown as stacked bars that show the mean with ± SD; n ≥ 3. The statistical significance is calculated using a two-way ANOVA and Tukey’s post hoc analysis. # *p* < 0.05, ## *p* < 0.01, ### *p* < 0.001 significant differences between probes treated with Au NPs, Pt-Au NPs, and Pt NPs, after exposure to NIR light and samples incubated only with examined particles. (**D**) After pre-gating on the singlets (based on their size and granularity), debris was excluded. Viable cells were chosen based on the parameters FSSC-A and SSC-A. The stimulatory expression of ICM is presented in the graphs as ΔMFI (mean fluorescence intensity).

As hepatoma cancer cells can modulate the antitumour immune response in direct contact with immune cells, we measured the surface expression of stimulatory (CD70, TNFRSF9, ICOS-L, and OX40-L) and suppressive (PD-L1, PD-L2, and HVEM) immune checkpoint molecules (ICMs) on the tumour cells after the 48 h of continuous incubation with examined NPs (55.6 µg/mL) and subsequently NIR exposition (Figure 7D and Figure 8D).

It is of note that exclusive NP treatment did not cause any substantial changes in the surface expression of the analysed stimulatory or suppressive ICMs. However, NIR irradiation alone or in combination with NP incubation resulted in alterations of the expression of PD-L1 and PD-L2 (Figure 7), as well as CD70, Ox40-L, and TNFRSF9 (Figure 8). This was most pronounced again in HepG2 cells.

Generally, as for HepG2 cells, a high dynamic of ICM expression could be observed after the combined treatment with Pt-Au NPs and NIR irradiation.

## 3. Discussion

**Figure 9:**
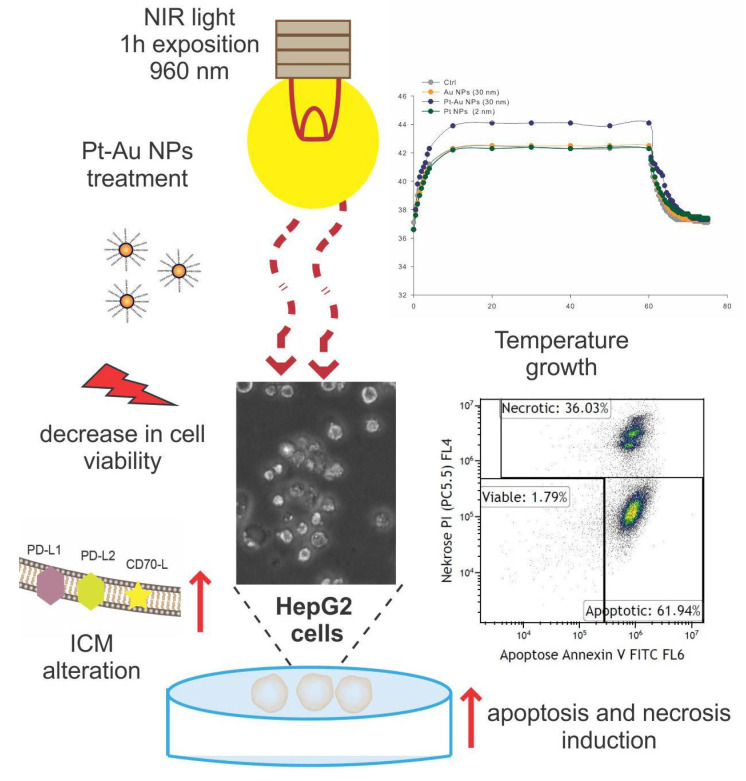
Model of Pt-Au NP- induced cellular effects, highlighting our most important findings. Pt-Au NPs activated by near-infrared (NIR) irradiation at 960 nm induced a localized temperature increase of up to 44 °C. The synergistic effects of hyperthermia and Pt-Au NP treatment were most pronounced in the HepG2 cell line. Cytotoxicity assessments demonstrated that a combined treatment of hyperthermia and Pt-Au NPs significantly reduced cell viability compared to the treatment with Pt-Au NPs alone. Furthermore, Annexin V/PI double staining was performed to examine the modes of cell death triggered by the combined treatment. The percentage of apoptotic and necrotic HepG2 cells was three times higher after simultaneous NP treatment and hyperthermia compared to controls (red arrow goes up). Immune checkpoint molecule expression was also analysed, revealing that NIR irradiation alone or in combination with NP treatment altered the expression levels of PD-L1, PD-L2 (red arrow goes up), CD70, OX40-L, and TNFRSF9. These results indicate that, among the tested NPs, only Pt-Au NPs exhibited activation by hyperthermia and demonstrated significant cytotoxic effects in all three HCC cell lines, with HepG2 cells showing the most robust response.

In the present work, we tested the hypothesis that hepatocellular carcinoma cells, after treatment with noble metal-based particles in combination with NIR-based hyperthermia, are affected more than these two techniques applied separately. The obtained data confirm and expand our previous work [36], in which we proved that Pt-containing nanoparticles are an interesting tool for improving the selectivity of anticancer therapies against HCC. Here, we additionally found that the cytotoxicity of Pt-Au NPs may be enhanced by NIR irradiation. These results are consistent with the temperature increase in cells growing medium that we observed after the exposition to NIR light only in samples containing Pt-Au particles (Figure 9). Moreover, all investigated hepatoma cancer cells displayed decreased metabolic activity toward a combination of NIR light and Pt-Au NP treatment. When we performed analyses of the morphology alterations of hepatocellular carcinoma cell lines after NP treatment combined with NIR irradiation, the changes in cellular shape and size were evident only for HepG2 cells. These observations are in line with the analysed cell death forms and revealed the supportive effect of NIR irradiation on Pt-Au NPs’ cytotoxicity and cell death induction in hepatocellular carcinoma cultures. In parallel, we found that a combination of NIR irradiation and incubation with Pt-Au NPs affected the expression of ICMs mainly on HepG2 cells.

Hyperthermia is the oldest approach in the fight against cancer [37]. However, different heating methods, e.g., microwave heating, warm bath heating, ultrasound, and NIR light, have different outcomes [38]. The therapeutic properties of HT have recently been improved by the establishment of novel drug delivery systems as a component of the combination therapy. Mild local HT can be used to enhance tissue perfusion and local drug release in tumour treatment. For example, ThermoDox^®^ (Celsion Corporation), a low temperature-sensitive liposome (LTSL) formulation, has completed its phase III clinical study in combination with standardized radiofrequency ablation in primary liver cancer [39]. Keeping in mind the common application of nanomaterials in biomedicine, it is important to understand how NPs influence patient responses in therapeutic settings of HT [11]. As a proof, the nanotechnology and thermotherapy fields have experienced exponential growth in the last 15 years, and with it, many data have been published on the effect of NPs on HT [40].

When the interactions between NIR light and NP application are considered, there is much scientific proof showing that the composition and design of NPs play a crucial role in determining how these two techniques affect toxicity. Furthermore, factors such as size, shape, and surface coating can influence the conversion of NIR light, as well as the subsequent biological effects [41]. Further, structural parameters of nanoparticles affect their toxicity in biomedical applications [42]. NPs are part of advances in targeted therapies used to increase the efficacy of multimodal antitumour therapies [43].

In the current study, we used three types of NPs that contained Pt and Au ions. However, it is challenging to define how NIR light can influence nanoparticle toxicity and why only Pt-Au core shells trigger a temperature increase in cellular growth conditions. The obtained results revealed that a temperature increase was induced only by Pt-Au core shells, even though we did not observe a maximum absorption for any of the tested nanoparticles. We speculate that this could be due to localized surface plasmon resonance (LSPR) observed for different nanoparticles. Hossain et al. [44] mentioned that the LSPR of gold NPs depends on the particle diameter. For example, 10 nm particles have a maximum LSPR of 520 nm, while 100 nm particles have a maximum LSPR of 580 nm. Nonetheless, it is possible to adjust the LSPR wavelength of NPs to be near NIR. This is a way of controlling different Au NPs, such as gold nanorods, nanocages, and nanoshells, which are applied to in vivo tumour models as photoacoustic imaging agents due to their LSPR-induced effects around NIR [44]. Salimi et al. showed that, due to the surface plasmon resonance, some Au NPs after exposure to NIR light can convert light energy into heat, causing localized hyperthermia (typically 42–45 °C) [45]. Because of the photocatalytic reactions, this phenomenon leads to the conversion of benign compounds into toxic ones and may impact the overall toxicity profile of NPs in a biological system. It may be an explanation as to why all investigated liver cancer cell lines displayed an improved sensitivity towards a combination of Pt-Au NP treatment and NIR light irradiation. The MTT assay that we used as an approach for viability analysis measures the mitochondrial activity of cells, and heat stress is one of the factors causing mitochondrial dysfunction [46]. Another detrimental factor of mitochondrial homeostasis is the generation of reactive oxygen species (ROS), mostly during respiratory chain reactions. Our previous work showed that the level of free radicals produced by Pt-Au NPs was two times higher compared to ultrasmall Pt NPs after 48 h of incubation in HepG2 cell culture [36]. This process can induce oxidative stress and, under conditions of elevated temperature, leads to protein denaturation, disruption of the cellular membrane, and damage to cellular organelles [47]. We conclude that these stressors triggered the changes of cellular membrane and caused the alteration of the morphology of cells.

The interaction between near-infrared light and nanoparticles has been a focal point of many studies, especially regarding the modulation of apoptosis [48,49]. Apoptosis, as one of the best described types of programmed cell death forms, is regulated by a complex network of molecular events, which involve the expression of different proapoptotic and antiapoptotic proteins, the externalization of phosphatidylserine, or the formation of apoptotic bodies [50]. NIR light is commonly used in conjunction with nanoparticles for therapeutic purposes, including photothermal therapy and photodynamic therapy, which can induce apoptosis in targeted cells, particularly cancer cells.

Sahovaler et al. showed a strong correlation between NIR light irradiation and chlorin photosensitizer assembled NPs, which yielded significant apoptosis in 65.7% of Call-33 oral cavity squamous cell carcinoma. Additionally, up-conversion nanoparticles (UCNPs) with a core shell structure (NaYF4:Yb,Er,Nd@NaYF4:Yb,Nd) applied together with NIR light activated caspase-3 and induced apoptosis [51].

The results obtained in the present study strongly support the hypothesis that NIR-based hyperthermia after the treatment of cells with core shell Ps (here, Pt-Au NPs) is more sufficient in triggering the apoptosis. We observed greater changes in the cell membrane asymmetry in HepG2 cells than both factors applied separately. Such dramatic disturbances of membrane integrity (in the same experimental conditions) we did not observe either in the Hep3B or Huh-7D 12 cell lines. However, it should be mentioned that our data from the MTT assay showed a comparable fraction of stressed cells in all three examined hepatocellular carcinoma cell lines. Keeping in mind that during the MTT assay the directly measured activity of oxidoreductases localized mainly in mitochondria [46], we observed a 1.4-fold decrease in the metabolic activity of HepG2 cells (Figure S1A) but only after NIR-light irradiation. This indicates that mitochondria are highly involved in the toxicity mechanisms initiated by NIR light and NP treatment. However, to draw final conclusions, future in vitro and in vivo analyses must be performed according to the guidelines for cancer cell death [52]. Although a combination of HT and noble metal-based NPs significantly decreased the viability of various types of cancer cell lines, NIR light-based therapy might also be beneficial to alter the immune phenotype of tumour cells, as already demonstrated for HT [38]. It is important to note that the sensitivity of cells toward heat varies, depending on the tumour microenvironment. Factors such as pH or hypoxia decreased the amount of nutrients supplied to the cancer tissue due to its chaotic vascularization. Furthermore, insufficient blood supply to tumour tissue reduces heat dissipation, resulting in higher temperatures. Finally, HT can cause enhanced antigen presentation, including T cell recruitment and activation of macrophages [53,54]. HT (particularly when combined with RT or chemotherapy) has been shown to influence the immune system, affecting the anticancer immune response [10] also in abdominal tumours [55,56]. In fact, immunogenic cell death can be activated in colorectal cancer cells by a combination of RT with graphene-induced HT [57] or conventional heat application [58].

It is worth emphasizing that anticancer therapeutics affect not only cancer cells but also the surrounding non-cancerous cells within the tumour microenvironment (TME). These include immune cells, stromal cells, and various signaling molecules [59]. Consequently, a range of responses can be triggered under these conditions. For instance, some drugs can enhance the immune system’s response against cancer, leading to the expression of stimulatory molecules that activate and recruit immune cells to attack the tumour [60]. Simultaneously, cancer cells can adapt to the stress conditions induced by treatment by upregulating inhibitory molecules. These include immune checkpoints such as PD-L1 and CTLA-4, which enable cancer cells to evade the immune system by putting “brakes” on immune responses. Finally, antineoplastic therapies can also induce inflammation, further complicating the immune landscape. Inflammatory signals can result in the expression of both pro-inflammatory (stimulatory) and anti-inflammatory (inhibitory) molecules [60]. The last explanation was observed in HepG2 cells exposed to Pt-Au NPs and irradiated with NIR, which expressed both stimulatory and inhibitory molecules. We assume that a combination of NIR-based hyperthermia and Pt-Au NP treatment triggered inflammation effects, which could be observed as a high percentage of necrotic cells. When looking closely at immune stimulatory ICM, we found for the first time that NIR light-based HT alone and in combination with NPs induced increased expression of CD70 and OX40-L. In contrast, exclusive particle treatment did not cause any significant changes [61]. OX40, for instance, is involved in the production of proinflammatory cytokines and the expansion of T cells. However, clinical trials using anti-OX40 monoclonal antibodies have shown that the nonspecific nature of this immune activation makes it ineffective against low immunogenic tumours [62]. In our study, the investigated experimental conditions had the greatest impact on TNFRSF9 upregulation, which is a member of the tumour necrosis receptor superfamily and considered a costimulatory molecule that results in activation and survival in CD8+ T cells. Additionally, it is an inducible cell surface receptor that is primarily found on activated T cells. Currently, Utomilumab is the only mAb targeting TNFRSF on the market [63]. However, Utomilumab trials were temporarily halted due to the risk of liver toxicity. It was probably related to the fact that there is a direct correlation between the TNFRSF9-mediated pathogenesis from chronic hepatitis to hepatocellular carcinoma that Wang et al. observed in in Hepatitis B Virus-transgenic mice [64]. Immune checkpoint molecules, on the other hand, have been linked to a variety of immune surveillance, editing, and escape processes [65]. For example, tumour cells block T cell-mediated antitumour immune responses via the PD-L1/PD-1 axis which is an important regulator of T cell activation [66].

The immunomodulatory properties of NIR-based HT have been investigated in various in vitro, in vivo, and phase I–III studies [67]. For example, Yu et al. tried to combine immune checkpoint blockade, immunogenic cell death, photothermal therapy, and tumour targeting in one go in a liposomal system. They integrated IR780 (photothermal agent), folic acid-linked oxaliplatin prodrug (tumour targeting + ICD), BMS-1 (PD-L1 inhibitor), and lipids to form thermosensitive liposomes. The FOIB@Lip liposomes (including IR780, FA-OXA, and BMS-1) allowed for tumour accumulation through the EPR effect and, upon NIR laser irradiation, showed better immunogenicity and tumour inhibition compared to FOIB@Lip without laser irradiation [68].

The PD-1/PD-L1 blockade has been revolutionary in cancer immunotherapy, and it has been used in the treatment of numerous malignancies, including melanoma, hepatocellular carcinoma, breast cancer, and Hodgkin’s lymphoma [69]. For instance, anti-PD-1 immune checkpoint inhibitors, such as Nivolumab or Pembrolizumab, are already included in therapy for unresectable or metastatic head and neck squamous cell carcinoma [70]. However, only a minority of patients (20–30%) are estimated to show a positive response to PD-1/PD-L1 blockade therapy. Patients may also acquire resistance that could eventually lead to cancer progression in patients who have had a clinical response [71]. Some recent studies have shown that HT will up-regulate the PD-L1 expression in tumour cells, thus making the tumour microenvironment immunosuppressive [72]. As a result, blocking PD-1 on the surface of T cells while applying mild HT would also give a promising development. This hypothesis was supported by Li et al., who stressed that HT can create a tumour niche with a high expression of PD-L1 and lymphocyte infiltration, making the tumour more likely to respond to anti-PD1 therapy [22]. Similar rationales can be used in our study, which demonstrated that NIR-based HT in combination with Pt-Au NPs increased the expression of PD-L1 as well as PD-L2 in HepG2 cancer cells. Furthermore, when we compared the basal expression of these molecules among examined hepatoma cancer cell lines, we discovered that, for PD-L1 and HVEM, the direction of expression’s alterations (HepG2 > Huh-7D 12 > Hep3B) are consistent with the route of their sensitivity under the tested conditions. Intriguingly, in all performed experiments, we observed a similar way of response among the investigated hepatoma cell lines. Why do we notice such a strong diversity in the reaction of HepG2, Hep3B, and Huh-7D 12 cells to tested, metal-based nanoparticles and applied together with NIR-based hyperthermia?

First, the examined human hepatoma cancer cell lines are derived from different ethnic origins, which correspond to various numbers of chromosomes. For example, HepG2 cells contain an average of 55 (50–56) chromosomes per cell [73], while Hep3B or Huh-7D 12 [74] cells have 60 and 59, respectively. These alterations are a direct consequence of mutations in genes that control the stability of the cellular genome. For instance, Hep3B did not show the activation of p53 protein, a commonly known cell cycle regulator in cancer cells. On the other hand, the mutant p53 (Y220C) is present in the Huh-7D 12 cell line, while HepG2 cells express a wild type [75] of this protein. Significant diversity in the profile gene expression is related to the various stages of differentiation, observed among tested in vitro models of liver cancers.

HepG2 and Huh-7D 12 S cell lines retain much more hepatocyte-related features, while Hep3B cells show fibroblast-related features and express more mesenchymal proteins, indicative of epithelial to mesenchymal transition [76].

Finally, differentiated responses of the hepatoma cell lines triggered by the examined techniques could be connected to distinguished viral susceptibility. HepG2 cells are negative for hepatitis B virus (HBV), but Hep 3b and Huh-7D 12 cell lines are positive to HBV [77]. The vulnerability of HBV has been described as in line with the expression of the HBV X protein (HBx). Through the interaction with various cellular signaling pathways, such as NF-κB, MAPK, and PI3K/Akt, HBx mediates the resistance of tumour cells to chemotherapy by preventing drug-induced apoptosis [78]. Additionally, the HBV infection can upregulate ICM like PD-1/PD-L1, which can suppress the immune response against cancer. This mechanism can make tumours more resistant to immunotherapy and other treatments that rely on immune activation [79].

## 4. Materials and Methods

### 4.1. Reagents

The chemical reagents used were as follows: gold (III) chloride trihydrate (HAuCl_4_·3H_2_O), sodium hydroxide (NaOH), trisodium citrate dihydrate (C_6_H_9_Na_3_O_9_), sodium hydroxide (NaOH), sodium hexachloroplatinate hexahydrate (Na_2_PtCl_6_·6H_2_O), ascorbic acid (C_6_H_8_O_6_), HSPEG- COOH (5 kDa), and 3-(4, 5-dimethylthiazolyl-2)-2, 5-diphenyltetrazolium bromide (MTT assay) were purchased form Merck (St. Louis, MO, USA). Dulbecco’s Modified Eagle’s Medium (DMEM), fetal bovine serum (FBS), penicillin streptomycin, L-glutamine, and phosphate-buffered saline were from Thermo Fisher (Thermo Fischer Scientific, Waltham, MA, USA). Antibodies used for immune checkpoint molecule assay were obtained from BioLegend (San Diego, CA, USA). Tissue culture dishes were supplied by Costar (Corning Incorporated, New York, NY, USA). All other chemicals and solvents used in this study were of the highest analytical grade.

### 4.2. Synthesis and Characterization of Pt-, Au- and Pt-Au NPs

The synthesis of PtNPs with a diameter of 2 nm was performed following the method described by Islam et al. [67], with a modification involving a 10-fold increase in platinum concentration at the initial stage. A 5 mM solution of H_2_PtCl_6_·6H_2_O in water was prepared and placed in a two-neck round-bottom flask. After connecting the flask to a reflux condenser, the solution was stirred at 1000 rpm on a magnetic stirrer for 30 min at 100 °C. Subsequently, the reducing agent C_6_Na_2_O_6_ (48.5 mM) was added to the reaction mixture, maintaining the molar ratio of C_6_Na_2_O_6_ to Pt at 3.88 [80]. After the synthesis was complete, the mixture was cooled to room temperature, and the size and shape of PtNPs were analysed by HR-TEM microscopy (TALOS™ F200X, Thermo Fischer Scientific, Waltham, MA, USA) and an ICP-MS spectrometer (Elan DRC II, Perkin Elmer, Waltham, MA, USA).

The synthesis of gold NPs with a diameter of 30 nm was carried out using the Turkevich method, in which Au^3+^ ions are reduced using sodium citrate [81]. For this purpose, 4.95 mg of HAuCl_4_·3H_2_O was dissolved in 50 mL of deionized water. Since pH is the main factor determining the diameter of NPs [82], the solution was alkalized with 0.1 M NaOH to achieve a pH range of 3.5 to 6.0, which facilitates the formation of 30 nm gold nanoparticles. Then, after 30 min of heating at 95 °C and stirring at 600 rpm, 170 μL of 340 mM Na_3_Ct·2H_2_O was added to the solution as both a reducing and stabilizing agent. The synthesis was conducted under these unchanged conditions for 3.5 h. After the reaction was completed, the solution was cooled to room temperature. The size distribution (Z-average) and the zeta potential (ζ) of the synthesized particles were measured using dynamic light scattering (DLS, Zetasizer Nano ZS, Malvern Instruments, Malvern, UK) in 0.01 M phosphate buffer (pH 7) at 25 °C.

For the synthesis of Pt-Au nanoparticles, the previously obtained Au NPs were coated with a platinum layer. A platinum precursor in the form of Na_2_PtCl_6_·6H_2_O was used [83], and 32 mL of Au NP solution was utilized for each synthesis. The initial reaction step involved heating the mixture in a two-neck round-bottom flask connected to a reflux condenser for 10 min at 90 °C and stirring at 280 rpm. The platinum precursor solution was prepared by dissolving an appropriate amount of Na_2_PtCl_6_·6H_2_O in deionized water to a concentration of 0.001 M. During reaction optimization, the synthesis was performed by adding 0.24–4.30 mg of the salt to the mixture, corresponding to 0.08–1.49 mg of Pt. To ensure a homogeneous distribution of Pt atoms on the AuNP surface, the Pt precursor solution was added to the reaction mixture in four equal portions at 15 min intervals. As a reducing and stabilizing agent, a solution of 0.004 M ascorbic acid in deionized water was used. The reductant was introduced into the reaction mixture in eight equal portions, with each added at 30 min intervals. After the final addition of ascorbic acid, the synthesis continued under the same conditions (90 °C, 280 rpm) for another 30 min. Upon completion of the reaction, the mixture was cooled to room temperature, and the particles were analysed using the DLS technique as described earlier. Additionally, the number of platinum atoms on the AuNP surface was determined by ICP-MS spectrometry. Calculation of the platinum layer thickness was performed as follows: 1 mL of supernatant, obtained after centrifuging Pt-Au nanoparticles, was measured using ICP-MS, which allowed for the determination of Pt atoms on the surface of the gold nanoparticles. In parallel UV/Vis absorption spectra of Pt NP (7 mg/mL), Au NP (1 mg/mL), and Pt-Au NP (1 mg/mL) nanoparticles were measured on the UV/Vis Varian spectrophotometer (Markham, ON, Canada), with a wavelength range of 200–1000 nm.

In the final step of synthesis, all the investigated NPs were PEGylated by adding 15,000 molar excesses of HS-PEG-COOH and mixing for 15 min, 30 min, 45 min, 1 h, 6 h, 12 h, and 24 h to determine the minimal time required for successful PEGylation. Based on the results, a PEGylation duration of 30 min was selected as optimal for further experiments, including in vitro studies. The obtained pegylated nanoparticles were purified by centrifugation at 6500 rpm, then dispersed in ultrapure deionized water and quantified using the DLS technique. For the 2 nm platinum nanoparticles, purification was carried out with a Vivaspin^®^ 500, using a 100 kDa cut-off, at 8000 rpm for 10 min. It was observed that, in these conditions, any unconjugated platinum nanoparticles were able to permeate the membrane.

### 4.3. Cell Lines and Cultivation Conditions

Three different human hepatoma cancer cell lines, Hep3B (Hep3B, ATCC, Manassas, VA, USA), HepG2 (Hep G2, ATCC, Manassas, VA, USA), and Huh-7D 12 cells (Huh-7D 12; Merck, Darmstadt, Germany) were grown at 37 °C in 5% CO_2_ and 90% humidity under sterile conditions. All examined cell lines were cultured in Dulbecco’s modified Eagle’s medium containing GlutaMAX (Thermo Fisher Scientific, Waltham, MA, USA) supplemented with 10% fetal bovine serum (Thermo Fisher Scientific, Waltham, MA, USA), 100 μg/mL streptomycin, and 100 U penicillin (Merck, Darmstadt, Germany). Cell lines were passaged twice per week with Trypsin (Thermo Fisher Scientific, Waltham, MA, USA) for a maximum of 15 passages. All cell lines tested negative for mycoplasma contamination using a MycoAlert TM Mycoplasma Detection Kit (Lonza, New York, NY, USA).

### 4.4. NIR Heating System Used for Hyperthermia Treatment of Hepatoma Tumour Cells

**Figure 10:**
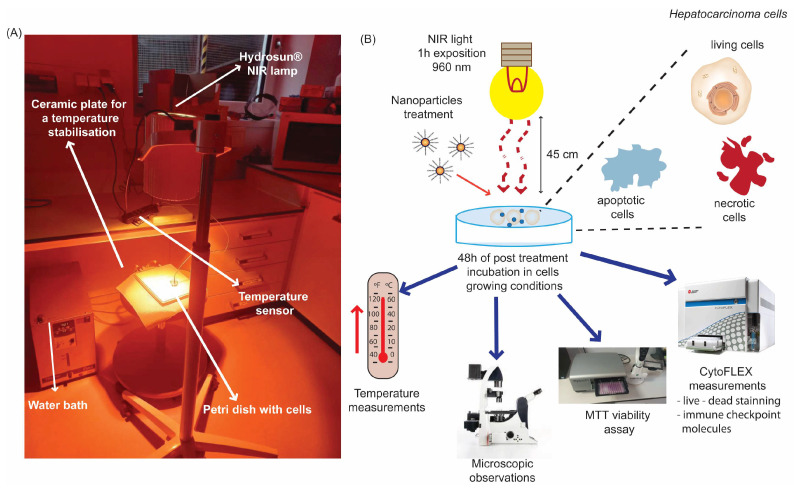
Graphical illustration of the experimental setup. (**A**) The heating device equipped with near infrared light (NIR, 960 nm, 1 h, 38.9 mW/cm^2^ applied by the Hydrosun^®^750 heating unit); (**B**) Outline of the analyses setup for the temperature alterations, viability changes, microscopic observation, cell death forms, and immune checkpoint molecules expression of hepatocellular carcinoma cells after treatments with NPs’ (55.6 µg/mL) and/or NIR-HT. Hepatocellular carcinoma cells were seeded one day prior to the treatment. The experiment consists of an NIR-HT arm, an NP treatment arm, and a combination of NIR-HT and NP treatment. For effective heating, the 3 cm^2^ Petri dish was placed on the NIR-exposed ceramic plate for 60 min. NP treatment was performed just before NIR exposure. Standard sampling was carried out in all arms on day 2 (48 h).

The heat treatment of the hepatoma tumour cells was performed in a self-designed heating item (Figure 10A) consisting of: (1) a water bath equipped with a temperature control unit type TS125 (H-Tronic GmbH, Hirschau, Germany), (2) a ceramic plate that allowed us to maintain a temperature suitable for cell growth conditions, (3) an NIR emitted lamp Hydrosun^®^750 (Hydrosun Medizintechnik, Müllheim, Germany), and (4) the temperature sensor Voltcraft K204 Thermometer (Voltcraft, Wollerau, Switzerland) [57,84].

To monitor the temperature increase in the cell growing conditions, the hepatoma cancer cells (3 × 10^5^ cells/well) were seeded in a 30 mm flat-bottom Petri dish. After 24 h, Pt, Pt-Au, and Au NPs in a concentration of 55.6 μg/mL were placed in the Huh-7D 12 cell culture. Next, cells were exposed to near-infrared irradiation (960 nm) for 1 h, with a distance of 45 cm between heat source and the Petri dish, at a power density of 38.9 mW/cm^2^ applied by Hydrosun^®^750. The temperature was monitored every 10 s with a Voltcraft K204 Thermometer (Voltcraft, Wollerau, Switzerland). A graphical illustration of the NIR-based heating item is shown in Figure 10A.

### 4.5. Growth Inhibition Assay

The growth inhibitory effect of noble metal-based NPs on Hep3B, HepG2, and Huh-7D 12 cells was evaluated by measuring cell viability using the MTT reduction assay. Briefly, cells were seeded in 100 μL of DMEM medium in 96-well transparent microtiter plates at a density of 7000 cells/well. After overnight incubation, the cells were exposed to a range of concentrations (0–139 μg/mL) of Pt, Au, and Pt-Au NPs with or without NIR exposition (NIR, 960 nm, 1 h, 45 cm, 38.9 mW/cm^2^ applied by Hydrosun^®^750). The cells were then grown at 37 °C under a 5% CO_2_ atmosphere for 48 h. Finally, the medium containing NPs was removed, and 50 μL of 50 mg% (*w*/*v*) MTT (Sigma-Aldrich, Darmstadt, Germany) in DMEM without phenol red medium was added to each well [85]. Subsequently, incubation continued for an additional 3 h, at 37 °C, for the formation of the formazan particles, which were dissolved by the addition of 100 μL of DMSO. Finally, the absorbance was read at 580 nm (analytical wavelength) and 720 nm (reference wavelength) using a Synergy2 plate reader (BioTek, Winooski, VT, USA).

### 4.6. Cellular Morphological Changes

In parallel with the readouts of the MTT viability assay, we examined the changes in cellular morphology after NP treatment using phase contrast microscopy. Such images were acquired by a LEICA microscope (Leica Microsystems, Wetzlar, Germany) equipped with a 20× inverted objective and a Digital Sight camera (Leica Microsystems, Wetzlar, Germany).

### 4.7. Cell Death Assay

The cell death forms of hepatoma tumour cells after NP treatment (55.6 µg/mL) and NIR exposure were analysed by multicolor flow cytometry, using Annexin V (AV)/propidium-iodide (PI) staining [38]. For this purpose, after the previously described settings of NP treatment (48 h) and NIR irradiation, the cells were harvested with trypsin and 1 × 10^5^ cells per 96-well were incubated with Annexin V (AV)/propidium iodide (PI) staining solution (1.0 µg/mL of PI and 0.5 μg/mL of FITC-labelled AV). Afterwards, the measurements were performed using a CytoFLEX S flow cytometer (the gating strategy is shown in Figure 5D). Cells that were positive for both AV and PI were defined as necrotic, and those that were positive for AV and negative for PI were qualified as apoptotic. The fraction of cells negative for both AV and PI was classified as viable. The gating strategy performed with Kaluza 2.0 (Beckman Coulter, Brea, CA, USA) is shown in Figure 5D.

### 4.8. Immune Checkpoint Molecule Expression Analysis

After treatments at 55.6 μg/mL of Pt, Pt-Au, and Au NPs for up to 48 h (with or without 1 h hyperthermia exposure), the tumour cells were harvested with trypsin, and 100,000 cells/well were incubated with 100 μL of the cell line-specific antibody staining solution (Figure 6A) for 30 min in the dark at 4 °C. To prevent an overlap of fluorescence peaks, all antibody concentrations (tested according to manufacturer’s instruction) were divided into two parts (Figure 6B) [86]. The mean fluorescence intensity (ΔMFI) of the stained samples was calculated by subtracting the fluorescence intensity of autofluorescence control samples (containing only the FACS buffer) from the fully stained samples. The samples were measured by flow cytometry (Beckman, Cytoflex S, Krefeld, Germany) and analysed using Kaluza 2.0 (Beckman Coulter, Brea, CA, USA). The details regarding the gating strategy and fluorescence readouts are provided in Figure 7D and Figure 8D.

### 4.9. Statistical Analysis

Data were expressed as the means ± SD. Normality analysis was performed using a Shapiro–Wilk test, and homogeneity of variance was assessed with the Brown–Forsythe test. The variables fulfilled the conditions of normality and homogeneity; thus, to indicate the statistically significant differences between the obtained results, a two-way ANOVA was performed, and Tukey’s test was used as post hoc analysis. For the paired variables during temperature measurements, we used a *t*-test with Bonferroni correction. All statistics were calculated using the STATISTICA program ver. 13.3. (StatSoft, Krakow, Poland). A *p* value of < 0.05 is considered significant [87].

## 5. Conclusions

In our experimental setting, we have shown for the first time that NIR-dependent hyperthermia enhanced the cytotoxic effect of Pt-Au-based NPs. Together with the upregulation of the stimulatory immune checkpoint molecules TNFRSF9 and CD70, which occurred after the tumour cells were treated with Pt-Au NPs and exposure to NIR, a higher fraction of apoptotic and necrotic tumour cells occurred. The HepG2 cells representing the HBV-negative hepatocellular carcinoma were the most sensitive to the examined treatments. All of the investigated liver cancer cells had significantly downregulated mitochondrial activity after combined treatments, even though only a minor impact on apoptosis and necrosis was observed. The preclinical data suggest that distinct NPs in combination with NIR could be beneficial in multimodal therapy settings in liver cancer, but only for selected patients.

## Data Availability

The datasets presented during the current study are available from the corresponding author on reasonable request.

## Supplementary Materials

The following supporting information can be downloaded at: https://www.mdpi.com/article/10.3390/ijms26041574/s1.

## Abbreviations

The following abbreviations are used in this manuscript:

APCs	Antigen-Presenting Cells
Au NPs	Gold Nanoparticles
AV	Annexin V
BTLA	B- and T-Lymphocyte Attenuator
CD70-L/R	Cluster of Differentiation 70
DC	Dendritic Cells
DLS	Dynamic Light Scattering
GO	Graphene Oxide
HBV	Hepatitis B Virus
HCC	Hepatocellular Carcinoma
HVEM	Herpesvirus Entry Mediator
HR-TEM	High-Resolution Transmission Electron Microscopy
HT	Hyperthermia
ICMs	Immune Checkpoint Molecules
IC-PMS	Inductively Coupled Plasma Mass Spectrometry
ICOS-L/R	Inducible T-cell COStimulator
mAb	Monoclonal Antibody
NIR	Near-Infrared Light
NPs	Nanoparticles
(NRs)	Nanoraspberries
OX40-L/R	Tumour Necrosis Factor Receptor Superfamily Member 4
PEG	Polyethylene glycol
PDI,	Polydispersity Index
PD/PD-L1, -L2	Programmed Cell Death 1/Ligand 1, 2
PI	Propidium Iodide
Pt NPs	Platinum Nanoparticles
Pt-Au NPs	Platinum Gold Nanoparticles
PT	Photothermal Therapy
TNFRSF9 (CD137)	Tumour Necrosis Factor Receptor Superfamily Member 9
ΔMFI	Mean Fluorescence Intensity
ζ	Zeta Potential

## References

[1] Thuluvath P.J., To C., Amjad W. (2021). Role of Locoregional Therapies in Patients with Hepatocellular Cancer Awaiting Liver Transplantation. Am. J. Gastroenterol..

[2] Jedrzak A., Grzeskowiak B.F., Golba K., Coy E., Synoradzki K., Jurga S., Jesionowski T., Mrowczynski R. (2020). Magnetite Nanoparticles and Spheres for Chemo- and Photothermal Therapy of Hepatocellular Carcinoma in vitro. Int. J. Nanomed..

[3] Kong F.H., Ye Q.F., Miao X.Y., Liu X., Huang S.Q., Xiong L., Wen Y., Zhang Z.J. (2021). Current status of sorafenib nanoparticle delivery systems in the treatment of hepatocellular carcinoma. Theranostics.

[4] Li L., Wang H. (2016). Heterogeneity of liver cancer and personalized therapy. Cancer Lett..

[5] Greten T.F., Sangro B. (2018). Targets for immunotherapy of liver cancer. J. Hepatol..

[6] Llovet J.M., Kelley R.K., Villanueva A., Singal A.G., Pikarsky E., Roayaie S., Lencioni R., Koike K., Zucman-Rossi J., Finn R.S. (2021). Hepatocellular carcinoma. Nat. Rev. Dis. Primers.

[7] Roayaie S., Obeidat K., Sposito C., Mariani L., Bhoori S., Pellegrinelli A., Labow D., Llovet J.M., Schwartz M., Mazzaferro V. (2013). Resection of hepatocellular cancer ≤2 cm: Results from two Western centers. Hepatology.

[8] Chen J., Yao Y., Mao X., Chen Y., Ni F. (2024). Liver-targeted delivery based on prodrug: Passive and active approaches. J. Drug Target..

[9] Subhan M.A., Parveen F., Filipczak N., Yalamarty S.S.K., Torchilin V.P. (2023). Approaches to Improve EPR-Based Drug Delivery for Cancer Therapy and Diagnosis. J. Pers. Med..

[10] Hader M., Frey B., Fietkau R., Hecht M., Gaipl U.S. (2020). Immune biological rationales for the design of combined radio- and immunotherapies. Cancer Immunol. Immunother..

[11] Bala V.M., Lampropoulou D.I., Grammatikaki S., Kouloulias V., Lagopati N., Aravantinos G., Gazouli M. (2023). Nanoparticle-Mediated Hyperthermia and Cytotoxicity Mechanisms in Cancer. Int. J. Mol. Sci..

[12] Roti Roti J.L., Kampinga H.H., Malyapa R.S., Wright W.D., vanderWaal R.P., Xu M. (1998). Nuclear matrix as a target for hyperthermic killing of cancer cells. Cell Stress Chaperones.

[13] Ademaj A., Veltsista P.D., Marder D., Halg R.A., Puric E., Brunner T.B., Crezee H., Gabrys D., Franckena M., Gani C. (2023). A patterns of care analysis of hyperthermia in combination with radio (chemo) therapy or chemotherapy in European clinical centers. Strahlenther. Onkol..

[14] Hildebrandt B., Wust P., Ahlers O., Dieing A., Sreenivasa G., Kerner T., Felix R., Riess H. (2002). The cellular and molecular basis of hyperthermia. Crit. Rev. Oncol. Hematol..

[15] Van der Zee J. (2002). Heating the patient: A promising approach?. Ann. Oncol..

[16] Wust P., Hildebrandt B., Sreenivasa G., Rau B., Gellermann J., Riess H., Felix R., Schlag P.M. (2002). Hyperthermia in combined treatment of cancer. Lancet Oncol..

[17] Chu K.F., Dupuy D.E. (2014). Thermal ablation of tumours: Biological mechanisms and advances in therapy. Nat. Rev. Cancer.

[18] Kase K., Hahn G.M. (1975). Differential heat response of normal and transformed human cells in tissue culture. Nature.

[19] Imashiro C., Takeshita H., Morikura T., Miyata S., Takemura K., Komotori J. (2021). Development of accurate temperature regulation culture system with metallic culture vessel demonstrates different thermal cytotoxicity in cancer and normal cells. Sci. Rep..

[20] Shiravand Y., Khodadadi F., Kashani S.M.A., Hosseini-Fard S.R., Hosseini S., Sadeghirad H., Ladwa R., O’Byrne K., Kulasinghe A. (2022). Immune Checkpoint Inhibitors in Cancer Therapy. Curr. Oncol..

[21] Morad G., Helmink B.A., Sharma P., Wargo J.A. (2021). Hallmarks of response, resistance, and toxicity to immune checkpoint blockade. Cell.

[22] Li Z., Deng J., Sun J., Ma Y. (2020). Hyperthermia Targeting the Tumor Microenvironment Facilitates Immune Checkpoint Inhibitors. Front. Immunol..

[23] Chong G., Zang J., Han Y., Su R., Weeranoppanant N., Dong H., Li Y. (2021). Bioengineering of nano metal-organic frameworks for cancer immunotherapy. Nano Res..

[24] Beik J., Abed Z., Ghoreishi F.S., Hosseini-Nami S., Mehrzadi S., Shakeri-Zadeh A., Kamrava S.K. (2016). Nanotechnology in hyperthermia cancer therapy: From fundamental principles to advanced applications. J. Control. Release.

[25] Yao Y., Zhou Y., Liu L., Xu Y., Chen Q., Wang Y., Wu S., Deng Y., Zhang J., Shao A. (2020). Nanoparticle-Based Drug Delivery in Cancer Therapy and Its Role in Overcoming Drug Resistance. Front. Mol. Biosci..

[26] Hwang S., Nam J., Jung S., Song J., Doh H., Kim S. (2014). Gold nanoparticle-mediated photothermal therapy: Current status and future perspective. Nanomedicine.

[27] Georgeous J., AlSawaftah N., Abuwatfa W.H., Husseini G.A. (2024). Review of Gold Nanoparticles: Synthesis, Properties, Shapes, Cellular Uptake, Targeting, Release Mechanisms and Applications in Drug Delivery and Therapy. Pharmaceutics.

[28] Sultana R., Yadav D., Puranik N., Chavda V., Kim J., Song M. (2023). A Review on the Use of Gold Nanoparticles in Cancer Treatment. Anti-Cancer Agents Med. Chem..

[29] Singh P., Pandit S., Mokkapati V., Garg A., Ravikumar V., Mijakovic I. (2018). Gold Nanoparticles in Diagnostics and Therapeutics for Human Cancer. Int. J. Mol. Sci..

[30] Almeida J.P., Figueroa E.R., Drezek R.A. (2014). Gold nanoparticle mediated cancer immunotherapy. Nanomedicine.

[31] Yu S., Xia G., Yang N., Yuan L., Li J., Wang Q., Li D., Ding L., Fan Z., Li J. (2024). Noble Metal Nanoparticle-Based Photothermal Therapy: Development and Application in Effective Cancer Therapy. Int. J. Mol. Sci..

[32] Porcel E., Liehn S., Remita H., Usami N., Kobayashi K., Furusawa Y., Le Sech C., Lacombe S. (2010). Platinum nanoparticles: A promising material for future cancer therapy?. Nanotechnology.

[33] Jain P.K., Huang X., El-Sayed I.H., El-Sayed M.A. (2008). Noble metals on the nanoscale: Optical and photothermal properties and some applications in imaging, sensing, biology, and medicine. Acc. Chem. Res..

[34] Depciuch J., Stec M., Klebowski B., Baran J., Parlinska-Wojtan M. (2019). Platinum-gold nanoraspberries as effective photosensitizer in anticancer photothermal therapy. J. Nanobiotechnol..

[35] Wawrowicz K., Majkowska-Pilip A., Gawel D., Chajduk E., Pienkowski T., Bilewicz A. (2021). Au@Pt Core-Shell Nanoparticle Bioconjugates for the Therapy of HER2+ Breast Cancer and Hepatocellular Carcinoma. Model Studies on the Applicability of (193m)Pt and (195m)Pt Radionuclides in Auger Electron Therapy. Molecules.

[36] Wawrowicz K., Majkowska-Pilip A., Szwed M., Zelechowska-Matysiak K., Chajduk E., Bilewicz A. (2022). Oxidative Status as an Attribute for Selective Antitumor Activity of Platinum-Containing Nanoparticles against Hepatocellular Carcinoma. Int. J. Mol. Sci..

[37] McCarthy E.F. (2006). The toxins of William B. Coley and the treatment of bone and soft-tissue sarcomas. Iowa Orthop. J..

[38] Sengedorj A., Hader M., Heger L., Frey B., Dudziak D., Fietkau R., Ott O.J., Scheidegger S., Barba S.M., Gaipl U.S. (2022). The Effect of Hyperthermia and Radiotherapy Sequence on Cancer Cell Death and the Immune Phenotype of Breast Cancer Cells. Cancers.

[39] Ma G.L., Lin W.F. (2023). Immune checkpoint inhibition mediated with liposomal nanomedicine for cancer therapy. Mil. Med. Res..

[40] Szwed M., Marczak A. (2024). Application of Nanoparticles for Magnetic Hyperthermia for Cancer Treatment-The Current State of Knowledge. Cancers.

[41] Abbasi R., Shineh G., Mobaraki M., Doughty S., Tayebi L. (2023). Structural parameters of nanoparticles affecting their toxicity for biomedical applications: A review. J. Nanopart. Res..

[42] Nazeer S.S., Saraswathy A., Nimi N., Santhakumar H., Radhakrishnapillai Suma P., Shenoy S.J., Jayasree R.S. (2023). Near infrared-emitting multimodal nanosystem for in vitro magnetic hyperthermia of hepatocellular carcinoma and dual imaging of in vivo liver fibrosis. Sci. Rep..

[43] Viktorsson K., Rieckmann T., Fleischmann M., Diefenhardt M., Hehlgans S., Rodel F. (2023). Advances in molecular targeted therapies to increase efficacy of (chemo)radiation therapy. Strahlenther. Onkol..

[44] Hossain M.I., Nanda S.S., Selvan S.T., Yi D.K. (2022). Recent Insights into NIR-Light-Responsive Materials for Photothermal Cell Treatments. Nanomaterials.

[45] Salimi M., Mosca S., Gardner B., Palombo F., Matousek P., Stone N. (2022). Nanoparticle-Mediated Photothermal Therapy Limitation in Clinical Applications Regarding Pain Management. Nanomaterials.

[46] Ghasemi M., Turnbull T., Sebastian S., Kempson I. (2021). The MTT Assay: Utility, Limitations, Pitfalls, and Interpretation in Bulk and Single-Cell Analysis. Int. J. Mol. Sci..

[47] Scutigliani E.M., Liang Y., Crezee H., Kanaar R., Krawczyk P.M. (2021). Modulating the Heat Stress Response to Improve Hyperthermia-Based Anticancer Treatments. Cancers.

[48] Chu S., Stochaj U. (2020). Exploring near-infrared absorbing nanocarriers to overcome cancer drug resistance. Cancer Drug Resist..

[49] Xie M., Gong T., Wang Y., Li Z., Lu M., Luo Y., Min L., Tu C., Zhang X., Zeng Q. (2024). Advancements in Photothermal Therapy Using Near-Infrared Light for Bone Tumors. Int. J. Mol. Sci..

[50] Szwed M., Kania K.D., Jozwiak Z. (2016). Assessment of pro-apoptotic activity of doxorubicin-transferrin conjugate in cells derived from human solid tumors. Int. J. Biochem. Cell Biol..

[51] Sahovaler A., Valic M.S., Townson J.L., Chan H.H.L., Zheng M., Tzelnick S., Mondello T., Pener-Tessler A., Eu D., El-Sayes A. (2024). Nanoparticle-mediated Photodynamic Therapy as a Method to Ablate Oral Cavity Squamous Cell Carcinoma in Preclinical Models. Cancer Res. Commun..

[52] Jeena M.T., Kim S., Jin S., Ryu J.H. (2019). Recent Progress in Mitochondria-Targeted Drug and Drug-Free Agents for Cancer Therapy. Cancers.

[53] Schildkopf P., Ott O.J., Frey B., Wadepohl M., Sauer R., Fietkau R., Gaipl U.S. (2010). Biological rationales and clinical applications of temperature controlled hyperthermia--implications for multimodal cancer treatments. Curr. Med. Chem..

[54] Knippertz I., Stein M.F., Dorrie J., Schaft N., Muller I., Deinzer A., Steinkasserer A., Nettelbeck D.M. (2011). Mild hyperthermia enhances human monocyte-derived dendritic cell functions and offers potential for applications in vaccination strategies. Int. J. Hyperthermia.

[55] Lukacsi S., Munkacsy G., Gyorffy B. (2024). Harnessing Hyperthermia: Molecular, Cellular, and Immunological Insights for Enhanced Anticancer Therapies. Integr. Cancer Ther..

[56] Liu P., Ye M., Wu Y., Wu L., Lan K., Wu Z. (2023). Hyperthermia combined with immune checkpoint inhibitor therapy: Synergistic sensitization and clinical outcomes. Cancer Med..

[57] Podolska M.J., Barras A., Alexiou C., Frey B., Gaipl U., Boukherroub R., Szunerits S., Janko C., Munoz L.E. (2020). Graphene Oxide Nanosheets for Localized Hyperthermia-Physicochemical Characterization, Biocompatibility, and Induction of Tumor Cell Death. Cells.

[58] Schildkopf P., Frey B., Ott O.J., Rubner Y., Multhoff G., Sauer R., Fietkau R., Gaipl U.S. (2011). Radiation combined with hyperthermia induces HSP70-dependent maturation of dendritic cells and release of pro-inflammatory cytokines by dendritic cells and macrophages. Radiother. Oncol..

[59] Zhao Y., Liu X., Liu X., Yu J., Bai X., Wu X., Guo X., Liu Z., Liu X. (2022). Combination of phototherapy with immune checkpoint blockade: Theory and practice in cancer. Front. Immunol..

[60] Zhao H., Wu L., Yan G., Chen Y., Zhou M., Wu Y., Li Y. (2021). Inflammation and tumor progression: Signaling pathways and targeted intervention. Signal Transduct. Target Ther..

[61] Zhuang J., Holay M., Park J.H., Fang R.H., Zhang J., Zhang L. (2019). Nanoparticle Delivery of Immunostimulatory Agents for Cancer Immunotherapy. Theranostics.

[62] Diehl L., den Boer A.T., Schoenberger S.P., van der Voort E.I., Schumacher T.N., Melief C.J., Offringa R., Toes R.E. (1999). CD40 activation in vivo overcomes peptide-induced peripheral cytotoxic T-lymphocyte tolerance and augments anti-tumor vaccine efficacy. Nat. Med..

[63] Jhajj H.S., Lwo T.S., Yao E.L., Tessier P.M. (2023). Unlocking the potential of agonist antibodies for treating cancer using antibody engineering. Trends Mol. Med..

[64] Wang J., Zhao W., Cheng L., Guo M., Li D., Li X., Tan Y., Ma S., Li S., Yang Y. (2010). CD137-mediated pathogenesis from chronic hepatitis to hepatocellular carcinoma in hepatitis B virus-transgenic mice. J. Immunol..

[65] Pardoll D.M. (2012). The blockade of immune checkpoints in cancer immunotherapy. Nat. Rev. Cancer.

[66] Lim Y.J., Koh J., Kim S., Jeon S.R., Chie E.K., Kim K., Kang G.H., Han S.W., Kim T.Y., Jeong S.Y. (2017). Chemoradiation-Induced Alteration of Programmed Death-Ligand 1 and CD8^+^ Tumor-Infiltrating Lymphocytes Identified Patients with Poor Prognosis in Rectal Cancer: A Matched Comparison Analysis. Int. J. Radiat. Oncol. Biol. Phys..

[67] Li Y., Zhang P., Tang W., McHugh K.J., Kershaw S.V., Jiao M., Huang X., Kalytchuk S., Perkinson C.F., Yue S. (2022). Bright, Magnetic NIR-II Quantum Dot Probe for Sensitive Dual-Modality Imaging and Intensive Combination Therapy of Cancer. ACS Nano.

[68] Yu J., He X., Wang Z., Wang Y., Liu S., Li X., Huang Y. (2021). Combining PD-L1 inhibitors with immunogenic cell death triggered by chemo-photothermal therapy via a thermosensitive liposome system to stimulate tumor-specific immunological response. Nanoscale.

[69] Parvez A., Choudhary F., Mudgal P., Khan R., Qureshi K.A., Farooqi H., Aspatwar A. (2023). PD-1 and PD-L1: Architects of immune symphony and immunotherapy breakthroughs in cancer treatment. Front. Immunol..

[70] Cohen E.E.W., Bell R.B., Bifulco C.B., Burtness B., Gillison M.L., Harrington K.J., Le Q.T., Lee N.Y., Leidner R., Lewis R.L. (2019). The Society for Immunotherapy of Cancer consensus statement on immunotherapy for the treatment of squamous cell carcinoma of the head and neck (HNSCC). J. Immunother. Cancer.

[71] Huang Q., Zhang H., Hai J., Socinski M.A., Lim E., Chen H., Stebbing J. (2018). Impact of PD-L1 expression, driver mutations and clinical characteristics on survival after anti-PD-1/PD-L1 immunotherapy versus chemotherapy in non-small-cell lung cancer: A meta-analysis of randomized trials. Oncoimmunology.

[72] Huang L., Li Y., Du Y., Zhang Y., Wang X., Ding Y., Yang X., Meng F., Tu J., Luo L. (2019). Mild photothermal therapy potentiates anti-PD-L1 treatment for immunologically cold tumors via an all-in-one and all-in-control strategy. Nat. Commun..

[73] Qiu G.H., Xie X., Xu F., Shi X., Wang Y., Deng L. (2015). Distinctive pharmacological differences between liver cancer cell lines HepG2 and Hep3B. Cytotechnology.

[74] Kasai F., Hirayama N., Ozawa M., Satoh M., Kohara A. (2018). HuH-7 reference genome profile: Complex karyotype composed of massive loss of heterozygosity. Hum. Cell.

[75] Mitchell J.K., Midkiff B.R., Israelow B., Evans M.J., Lanford R.E., Walker C.M., Lemon S.M., McGivern D.R. (2017). Hepatitis C Virus Indirectly Disrupts DNA Damage-Induced p53 Responses by Activating Protein Kinase R. MBio.

[76] Shi J., Wang X., Lyu L., Jiang H., Zhu H.J. (2018). Comparison of protein expression between human livers and the hepatic cell lines HepG2, Hep3B, and Huh7 using SWATH and MRM-HR proteomics: Focusing on drug-metabolizing enzymes. Drug Metab. Pharmacokinet..

[77] Knowles B.B., Howe C.C., Aden D.P. (1980). Human hepatocellular carcinoma cell lines secrete the major plasma proteins and hepatitis B surface antigen. Science.

[78] Sivasudhan E., Blake N., Lu Z., Meng J., Rong R. (2022). Hepatitis B Viral Protein HBx and the Molecular Mechanisms Modulating the Hallmarks of Hepatocellular Carcinoma: A Comprehensive Review. Cells.

[79] Zhang X., Tian D., Chen Y., Chen C., He L.N., Zhou Y., Li H., Lin Z., Chen T., Wang Y. (2021). Association of hepatitis B virus infection status with outcomes of non-small cell lung cancer patients undergoing anti-PD-1/PD-L1 therapy. Transl. Lung Cancer Res..

[80] Islam M.T., Saenz-Arana R., Wang H., Bernal R., Noveron J.C. (2018). Green synthesis of gold, silver, platinum, and palladium nanoparticles reduced and stabilized by sodium rhodizonate and their catalytic reduction of 4-nitrophenol and methyl orange. New J. Chem..

[81] Rahme K., Holmes J. (2015). Gold Nanoparticles: Synthesis, Characterization, and Bioconjugation.

[82] Tanaka M., Hayashi M., Roach L., Kiriki Y., Kadonosono T., Nomoto T., Nishiyama N., Choi J., Critchley K., Evans S.D. (2021). Synthesis of near-infrared absorbing triangular Au nanoplates using biomineralisation peptides. Acta Biomater..

[83] Gao Z., Ye H., Tang D., Tao J., Habibi S., Minerick A., Tang D., Xia X. (2017). Platinum-Decorated Gold Nanoparticles with Dual Functionalities for Ultrasensitive Colorimetric in Vitro Diagnostics. Nano Lett..

[84] Podolska M.J., Shan X., Janko C., Boukherroub R., Gaipl U.S., Szunerits S., Frey B., Munoz L.E. (2021). Graphene-Induced Hyperthermia (GIHT) Combined with Radiotherapy Fosters Immunogenic Cell Death. Front. Oncol..

[85] Valsalakumari R., Yadava S.K., Szwed M., Pandya A.D., Maelandsmo G.M., Torgersen M.L., Iversen T.G., Skotland T., Sandvig K., Giri J. (2021). Mechanism of cellular uptake and cytotoxicity of paclitaxel loaded lipid nanocapsules in breast cancer cells. Int. J. Pharm..

[86] Hader M., Streit S., Rosin A., Gerdes T., Wadepohl M., Bekeschus S., Fietkau R., Frey B., Schlucker E., Gekle S. (2021). In Vitro Examinations of Cell Death Induction and the Immune Phenotype of Cancer Cells Following Radiative-Based Hyperthermia with 915 MHz in Combination with Radiotherapy. Cells.

[87] Wigner P., Zielinski K., Michlewska S., Danielska P., Marczak A., Ricci E.J., Santos-Oliveira R., Szwed M. (2021). Disturbance of cellular homeostasis as a molecular risk evaluation of human endothelial cells exposed to nanoparticles. Sci. Rep..

