# Supplementary material for: Pt-Au Nanoparticles in Combination with Near-Infrared-Based Hyperthermia Increase the Temperature and Impact on the Viability and Immune Phenotype of Human Hepatocellular Carcinoma Cells
Source: Int J Mol Sci. 2025 Feb 13;26(4):1574. doi: 10.3390/ijms26041574 (PMC11855494; doi:10.3390/ijms26041574)
Supplement: Supplementary file 1 [file ijms-26-01574-s001.zip › ijms-3431153-supplementary.pdf]

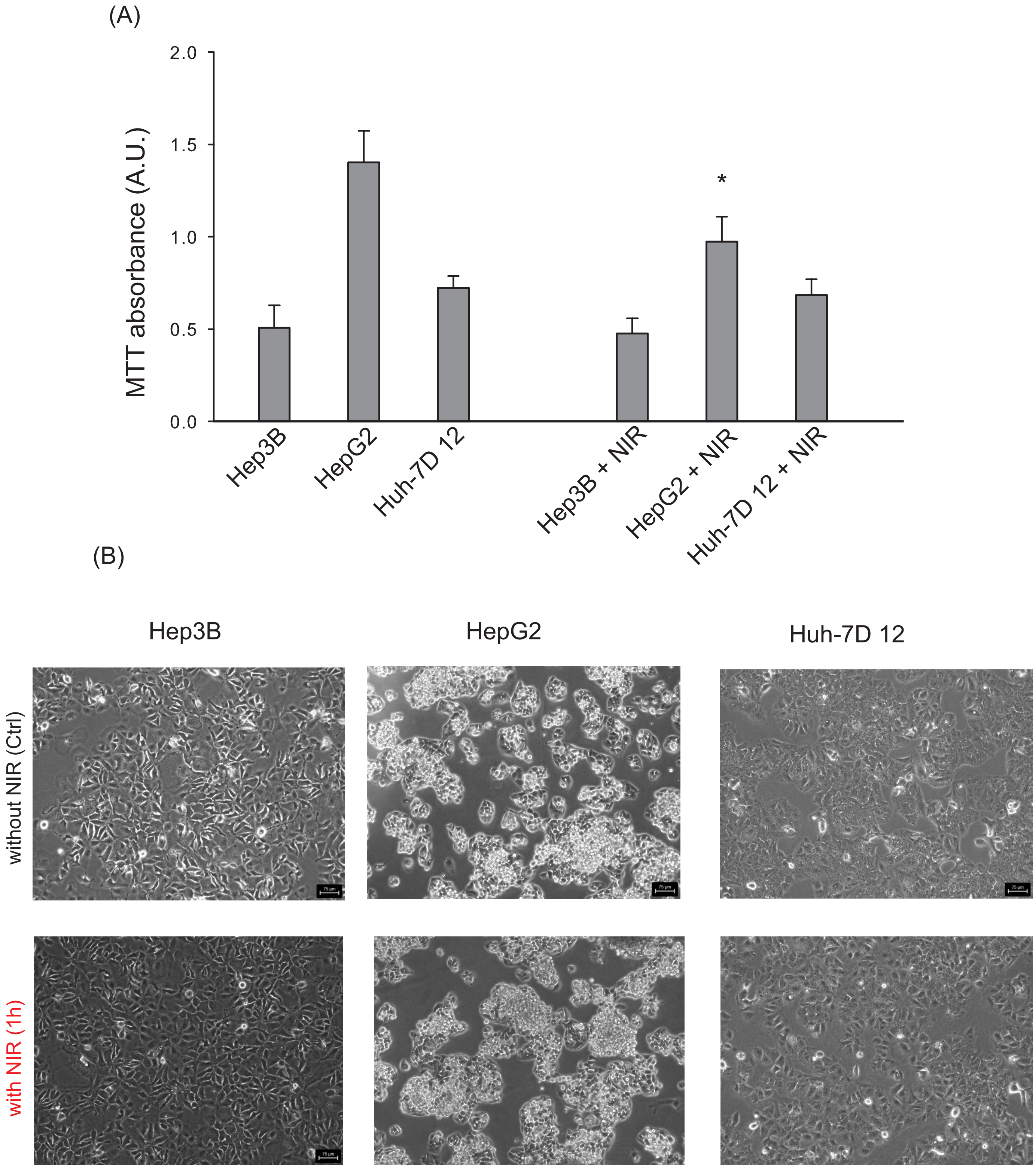

Figure S1. The response of the examined human hepatocellular carcinoma cell lines to NIR exposure. (A) The cytotoxicity of NIR irradiation by itself measured by the MTT assay. The data are shown as the absorbance mean values ± SD from three independent experiments, *$p<0.05$ the statistically significant changes (calculated using the t-test) between cells with and without NIR exposition (B) Inverted phase contrast microscopy images were obtained following treatment of Hep3B and Huh-7D 12 and HepG2 cells just after 1 hr of NIR exposition. The scale bar represents 75 μm.
